# Green spot in ‘WA 38’: Growth dynamics, vascular integrity and architecture, and a hypothetical mechanism

**DOI:** 10.1371/journal.pone.0351512

**Published:** 2026-07-10

**Authors:** Bruno Carra, Sara Serra, Moritz Knoche, David Rudell, Stefano Musacchi

**Affiliations:** 1 Tree Fruit Research and Extension Center, Washington State University, Wenatchee, Washington, United States of America; 2 Department of Horticulture, Washington State University, Pullman, Washington, United States of America; 3 The Pennsylvania State University, Department of Plant Science, University Park, Pennsylvania, United States of America; 4 Fruit Research and Extension Center, The Pennsylvania State University, Biglerville, Pennsylvania, United States of America; 5 United States Department of Agriculture, Tree Fruit Research Laboratory, Agricultural Research Service, Wenatchee, Washington, United States of America; United States Department of Agriculture, UNITED STATES OF AMERICA

## Abstract

‘WA 38’ is a cross of ‘Enterprise’ and ‘Honeycrisp’. It is susceptible to the surface disorder known as green spot (GS), which reduces its marketability. This study aimed to characterize fruit growth in ‘WA 38’, ‘Enterprise’, and ‘Honeycrisp’ and explore potential links to vascular integrity. Among the three cultivars, ‘WA 38’ exhibited the most rapid fruit growth, followed by ‘Enterprise’ and ‘Honeycrisp’. While fruit diameter was similar across cultivars, ‘WA 38’ apples were significantly longer. Allometric analysis revealed hyperallometric growth in the height of the calyx lobes and pedicel end region relative to diameter in all cultivars. Conversely, core height growth was hypoallometric. ‘WA 38’ showed faster calyx lobe elongation than either parent cultivar. However, in the pedicel end region, ‘Honeycrisp’ exhibited faster growth than ‘WA 38’. The positions of the vascular bundles were unusual across the three cultivars. Petal bundles were more peripheral than sepal bundles in ‘WA 38’, followed by ‘Enterprise’ and ‘Honeycrisp’. Acid fuchsin feeding pedicels of immature ‘WA 38’ fruit stained sepal and petal bundles and a dense network of minor veins. In mature fruit, staining was limited to petal and sepal bundles near the seed cavity. More petal bundles were stained in immature fruit than mature fruit, while sepal bundle staining remained constant. Notably, rupture of petal bundles occurred near the seed cavity. Green spot disorder did not consistently affect the calcium-to-dry-mass ratio. The xylem of ‘WA 38’ becomes dysfunctional, probably because of hyperallometric growth in height at the pedicel end and the unusual peripheral position of petal bundles. Although the total Ca/dry mass ratio of ‘WA 38’ appears sufficiently high, imbalances will likely arise between different Ca pools within the fruit.

## Introduction

‘WA 38’ is an apple cultivar released by the Washington State University Apple Breeding Program to growers in Washington State in 2017. The cultivar was marketed as Cosmic Crisp^®^ starting in 2019. The cultivar results from a cross between ‘Enterprise’ (maternal component) and ‘Honeycrisp’ (paternal component) [1]. ‘WA 38’ possesses key traits from both parents, such as red skin color from the maternal side [2] and texture from the paternal side [3,4]. Average fruit diameter and shape is 70–76 mm, “round to elongated” for ‘Enterprise’ [2], 70–90 mm, “oblate to roundly oblate” for ‘Honeycrisp’ [4], and 84 mm “round conical” for ‘WA 38’ [1]. However, under WA growing conditions, it is not uncommon to observe larger sizes (90 mm to 110 mm) for both ‘Honeycrisp’ [5] and ‘WA 38’ apples [6,7]. While ‘Honeycrisp’ is highly susceptible to bitter pit (BP) [8–10], ‘WA 38’ can develop “green spot” (GS) [11,12], a disorder that has not been observed in either parent.

While bitter pit, which is characterized by brown corky depressions concentrated in the calyx end of the ‘Honeycrisp’ apples [13–15], can be reduced by calcium application during the growing season [16,17], GS is not impacted, even by multiple calcium sprays, under Washington conditions (unpublished data). Reduced GS incidence was observed when ‘WA 38’ apples were enclosed in double-layered paper bags early in the season (at 44 DAFB and 60 DAFB) [11]. However, ten percent shading of ‘WA 38’ trees at 44–45 DAFB trees (pearl color net) had no impact on GS. The mechanism underlying these effects is unknown. Rootstock influences GS incidence, with more vigorous rootstocks (e.g., G.41) developing higher incidence than less vigorous rootstocks (e.g., M9-Nic29) [11,18]. Studies of xylem function in apple have shown that different cultivars may exhibit xylem dysfunction at different times during development, affecting fruit nutrient concentrations and, therefore, susceptibility to bitter pit [19,20]. Dražeta and colleagues (2004) concluded that BP symptoms were linked to xylem disruption caused by excessive fruit growth and expansion [20], potentially limiting Ca transport to the distal portion of the fruit [14]. This topic was most recently reviewed by Griffith and Einhorn (2022) [21]. The authors highlighted the correlation between BP incidence, the selection of large-size, domesticated apple cultivars over the last decades, and the roles of auxin deficiency and other hormonal imbalances in xylem dysfunction. These studies gave us reason to consider ‘WA 38’ might have a genetic predisposition to growth stress, which can result in vasculature disruption in the flesh underneath lenticels, manifesting as corky-dry areas similar to bitter pit. Previous investigations have reported a close association between severe GS and lenticels [11,12]. Unfortunately, definitive and causal evidence is lacking. To our knowledge, there is no information on fruit growth rates, vascular integrity, or surface characteristics for ‘WA 38’. Such information, however, would be helpful for better understanding the causes and mechanisms underlying the GS disorder in ‘WA 38’ and similar disorders in related apple cultivars [22]and for developing and implementing effective mitigation strategies that minimize the proportion of unmarketable apples in the orchard.

The objective of our study was to establish the fruit growth characteristics of ‘WA 38’ and to determine how these may be related to vascular integrity and the occurrence of GS. We used bagged ‘WA 38’ fruit as a positive control since bagging consistently reduced and prevented formation of GS [11,12]. We expected the high growth rates to impair vascular integrity and Ca import in ‘WA 38’.

## Materials and methods

### Plant material

In 2024, developing ‘WA 38’ apples and its parental cultivars—’Enterprise’ (maternal) and ‘Honeycrisp’ (paternal)—were sampled alongside mature fruit from a diverse set of randomly selected locally available apple cultivars: ‘Ambrosia’, ‘Earlygold’, ‘Gala’, ‘Granny Smith’, ‘Idared’, ‘Jonastar’, ‘Malus 22’, ‘Newtown Pippin’, ‘Cripp’s Pink’, ‘Delicious’, ‘September Wonder’, and ‘WA 64’. All samples were collected from experimental orchards at the Washington State University Sunrise Research Orchard (SRO) in Rock Island, WA, USA (47°18′N, 120°07′W; elevation 267 m).

‘WA 38’ trees, planted in 2013, were grafted onto G.41 rootstocks. ‘Honeycrisp’ (planted in 2014) and ‘Enterprise’ (planted in 2008) were grafted onto M.9-T337. The additional cultivars were grafted on various rootstocks as follows: ‘Ambrosia’/EMLA26, ‘Earlygold’/EMLA7, ‘Gala’/G.41, ‘Granny Smith’/unknown, ‘Idared’/EMLA7, ‘Jonastar’/EMLA26, ‘*Malus*22’/B118, ‘Newtown Pippin’/B118, ‘Cripp’s Pink’/G.11, ‘Delicious’/unknown, ‘September Wonder’/EMLA7, and ‘WA 64’/G.41.

A population of 608 ‘WA 38’ fruit were bagged 48 days after full bloom (DAFB), across 10 experimental trees (avg. 61 bags tree^-1^) grafted on G.41 and randomized across 5 rows. Each cluster was singularized by hand thinning to a single fruit per cluster. The same 2-layer apple bags used in our earlier study (Kobayashi Bag; Nagano, Japan) [11] were used. Another set of 10 trees of ‘WA 38’/G.41 were also hand-thinned to a single fruit per cluster and remained without bag (659 total fruit, avg. 66 apples tree^-1^). Bagging was used as an additional control [11].

Orchard management was carried out in accordance with current regulations for integrated fruit production and standard practices in Washington State (USA).

Fruit intended for allometric evaluation was stored in a regular cold storage at 1 ± 1°C for up to 2 days before analysis. Apples harvested for general GS grading were stored for a maximum of 35 days. Earlier studies established no significant change in GS incidence or severity during storage [11].

### Fruit growth, allometry and vasculature

**Fig 1:**
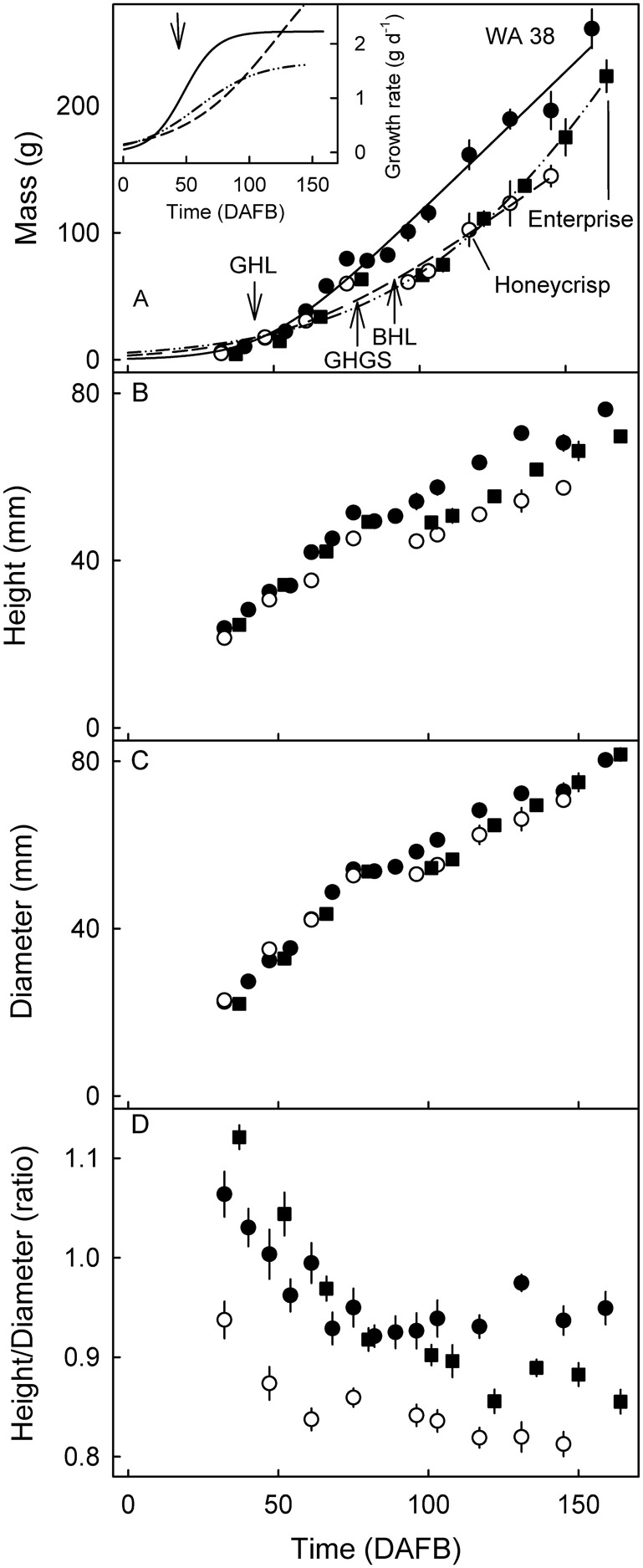
Time course of fruit growth (0 to 160 DAFB) of ‘WA 38’ (closed circles), ‘Honeycrisp’ (open circles) and ‘Enterprise’ (closed squares). Fruit mass **(A)**, height **(B)**, equatorial diameter **(C)** and height/diameter ratio vs. time **(D)**; time scale in days after full bloom (DAFB). Inset in A: Growth rate in fruit fresh mass vs. time, solid line for ‘WA 38’, dashed line for ‘Honeycrisp’ and double-dot-dashed line for ‘Enterprise’. Arrows in A indicate time of first symptoms of lenticels with green halo (GHL) at 47 DAFB, of developing green spots (GHGL) at 80 DAFB and of a severe green spot that is associated with necrosis (BHL) in ‘WA 38’. Data represent means ± SE.

**Fig 2:**
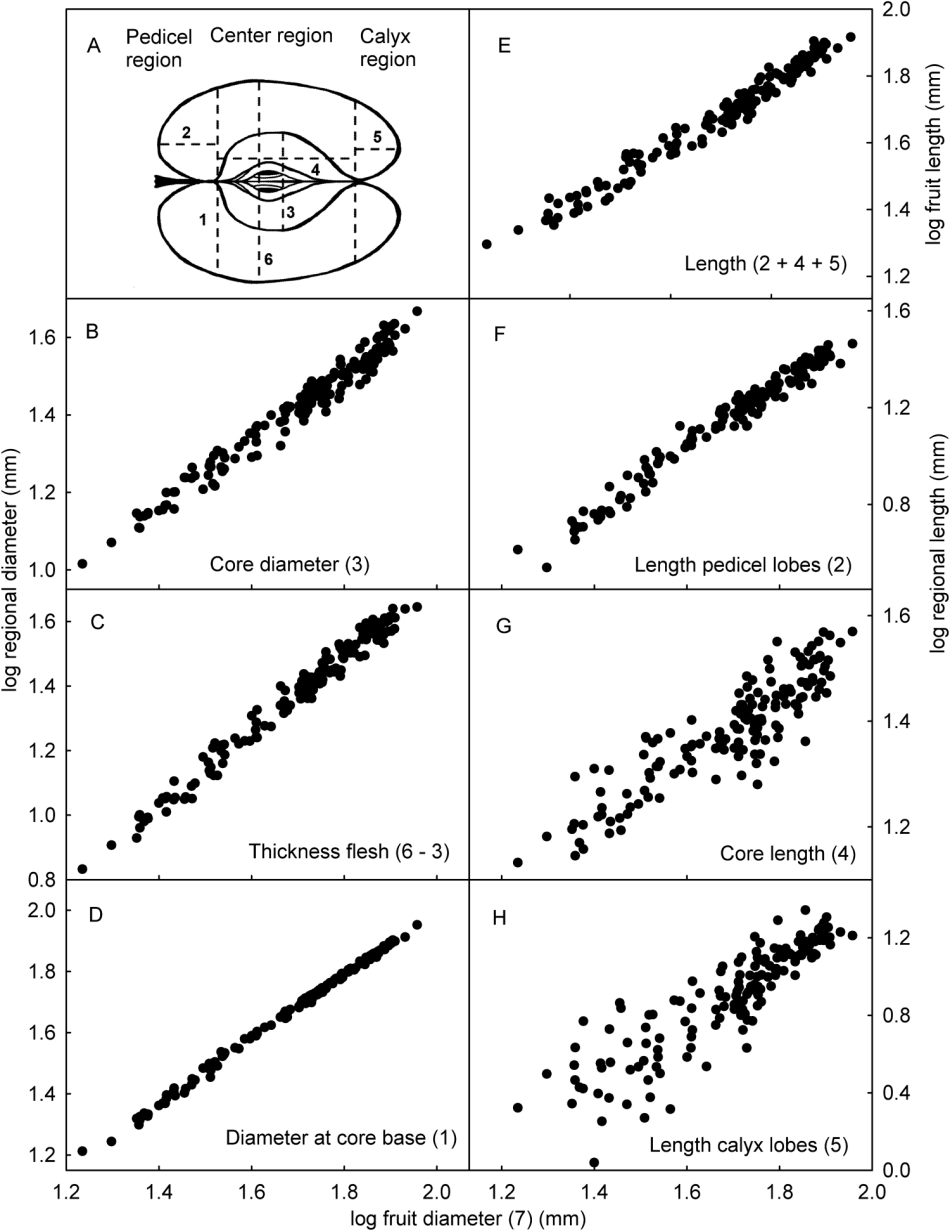
Allometric relationships between different longitudinal measures of developing ‘WA 38’ apples. **(A)** Sketch of longitudinal section and dimensions identified. **(B-H)** Allometric relationships between the fruit center diameter and the maximum core diameter **(B)**, the thickness of the flesh **(C)**, the fruit diameter at the core base **(D)**, total fruit length **(E)**, the length of the pedicel lobs **(F)**, the core length **(G)** and the length of the calyx lobs **(H)**. All dimensions are log transformed. For regression equations see Table 1.

Fruit growth was monitored in developing ‘WA 38’, ‘Honeycrisp’ and ‘Enterprise.’ Fruit were sampled in weekly or biweekly intervals beginning at 32–35 DAFB. Commercial maturity was reached at about 145 DAFB for ‘Honeycrisp’, 159 DAFB for ‘WA 38’, and 162 DAFB for ‘Enterprise.’ Fruit length and equatorial diameters were determined, and the length/diameter ratio was calculated. Fruit fresh mass was calculated using a regression equation for the relationship between fruit fresh mass (Fig 1) and fruit volume established on a separate data set obtained in the same season in the same orchard (S1 Fig). Allometric relationships between various dimensional measures of fruit growth and the increase in equatorial diameter in developing ‘WA 38’, ‘Honeycrisp’ and ‘Enterprise’ apples were analyzed [23,24]. Fruit were cut longitudinally along the calyx-pedicel axis. Calibrated photographs were prepared using a stereomicroscope (SMZ18; Nikon) or a digital camera (EOS 70D DSLR; Canon, Tokyo, Japan) mounted on a photo stand (CS42K 42″ Pro-Duty Copy Stand; Smith Victor, Chicago, ILL, USA). The calibrated images were analyzed using image analysis software (NIS-Elements BR 5.30.03 software, Nikon). The following dimensions were quantified: fruit diameter, fruit length, length of calyx lobes, length of core, length of pedicel lobes, diameter at core base, diameter of core, and thickness of flesh (Tc) (see Fig 2A and 2H). In addition, the above dimensions were also quantified for mature bagged ‘WA 38’, non-bagged asymptomatic fruit (no GS) and non-bagged symptomatic fruit having green spots (stage GHGS = lenticels with initial green spot formation). The number of replicates was 25.

**Table 1:** Estimates of regression parameter of the relationships of log transformed equatorial fruit diameter (in mm) and various dimensions (in mm) of developing ‘WA 38’, ‘Honeycrisp’, and ‘Enterprise’ apples. For a sketch of the dimensions measured on longitudinal sections of the apple fruit (see Fig 2A). The data are plotted for ‘WA 38’ in Fig 2, for ‘Enterprise’ in S3 Fig, and for ‘Honeycrisp’ in S4 Fig. Slope terms of regression equations represent differential growth ratios [23]. A slope >1 implies growth more rapid in y than in x dimension (hyperallometric), a slope <1 growth less rapid in y than in x dimension (hypoallometric), a slope = 1 growth equally rapid in y and x dimension (isometric). Growth in all dimensions (y-variables) is expressed relative to growth in diameter (x-variable).

Cultivar	Parameter (mm)	Coefficients of linear regression equation		
		Slope ± SE	Intercept ± SE	*r* ^2^
‘**WA 38**’	**Log length**	0.91 ± 0.02	0.13 ± 0.01	0.97***
	**Log length calyx lobes**	1.48 ± 0.11	−1.60 ± 0.11	0.78***
	**Log core length**	0.55 ± 0.02	0.46 ± 0.04	0.80***
	**Log length pedicel lobes**	1.31 ± 0.02	−1.06 ± 0.03	0.97***
	**Log diameter core base**	1.05 ± 0.00	−0.10 ± 0.01	1.00***
	**Log core diameter**	0.86 ± 0.01	−0.04 ± 0.02	0.97***
	**Log thickness flesh**	1.17 ± 0.01	−0.61 ± 0.02	0.98***
‘**Honeycrisp**’	**Log length**	0.89 ± 0.01	0.11 ± 0.03	0.98***
	**Log length calyx lobes**	1.32 ± 0.06	−1.29 ± 0.11	0.83***
	**Log core length**	0.40 ± 0.03	0.63 ± 0.05	0.69***
	**Log length pedicel lobes**	1.61 ± 0.03	−1.67 ± 0.06	0.96***
	**Log diameter core base**	1.11 ± 0.01	−0.20 ± 0.01	1.00***
	**Log core diameter**	0.85 ± 0.02	−0-02 ± 0.04	0.95***
	**Log thickness flesh**	1.18 ± 0.02	−0.64 ± 0.04	0.96***
‘**Enterprise**’	**Log length**	0.79 ± 0.01	0.33 ± 0.02	0.98***
	**Log length calyx lobes**	1.06 ± 0.03	−0.85 ± 0.05	0.93***
	**Log core length**	0.47 ± 0.02	0.58 ± 0.04	0.82***
	**Log length pedicel lobes**	1.27 ± 0.02	−1.05 ± 0.04	0.97***
	**Log diameter core base**	1.14 ± 0.01	−0.26 ± 0.01	1.00***
	**Log core diameter**	0.86 ± 0.02	−0.02 ± 0.03	0.97***
	**Log thickness flesh**	1.18 ± 0.02	−0.66 ± 0.03	0.98***

The slopes of all relationships were significant at *p* ≤ 0.001 (***).

### Position of petal and sepal bundles

The position of petal and sepal bundles relative to the center of the core was determined in the equatorial plane of mature ‘WA 38’, and its parents ‘Enterprise’ and ‘Honeycrisp’. In addition, a collection of cultivars comprising ‘Ambrosia’, ‘Earlygold’, ‘Gala’, ‘Granny Smith’, ‘Idared’, ‘Jonastar’, ‘*Malus* 22’, ‘Newton Pippin’, ‘Cripp’s Pink’, ‘Delicious’, ‘September Wonder’, and ‘WA 64’ was sampled at maturity for comparison. Briefly, apples were cut into slices approximately 5 mm thick using a multi-blade cutter (model 56750−2; Nemco Food Equipment, Hicksville, Ohio, USA) or a sharp knife. The equatorial slice with the largest diameter was selected and placed on a flatbed scanner (Plustek OpticSlim 1180, Plustek, Taipai, Taiwan). Calibrated scans were analyzed to quantify fruit diameter and the number and radial distance of each petal and sepal bundle from the core using image analysis software (NIS-Elements, Nikon). The radial distance of the vascular bundles from the core was determined as the distance between the center of the core and the individual petal or sepal bundle. The total number of individual fruit replicates was 12 per cultivar.

### Functionality of petal and sepal bundles

The functionality of vascular bundles was established in immature and mature ‘WA 38’ using acid fuchsin feeding [25]. For the immature stage, fruit was sampled from secondary-bloom fruit, with a mean fresh mass of 45.3 ± 4.7 g per fruit. This stage was equivalent to fruit sampled from the primary bloom at 65 DAFB. Fruit of the mature stage was sampled at 144 DAFB. In a second experiment on ‘WA 38’ conducted at DAFB 153 and 154, the effect of bagging on the functionality of vascular bundles was investigated. For sampling, representative fruit were selected in the orchard and submerged in deionized water. The fruit were detached from the spurs by cutting pedicels underwater. Since the xylem is under negative pressure, cutting at the ambient atmosphere would result in air entry into the xylem, embolism and artifacts due to blockage of the xylem [26]. Tygon tubing (inner diameter 1.1 or 2.2 mm, 100 mm length) was mounted on the fruit pedicels using silicone rubber (Dowsil^TM^ SE 9186 clear, Krayden, CO, USA). The tubing was filled with 0.1% acid fuchsin solution (Millipore Sigma, Rockville, MD, USA). The fruit were incubated at 24.0 ± 0.5°C and 45 ± 5% relative humidity for about 22 h. Tubes were refilled as needed during the course of the incubation. After 22 h, the amount of dye uptake was recorded as the length of movement of the meniscus of the fuchsin solution in the tubing (mm) multiplied by the cross-sectioning area of the tubing (mm^2^). After removing the remaining dye solution and the tubing, the fruit were sectioned transversely in the equatorial plane. The number of non-stained and stained petal and sepal bundles was quantified (see S2 Fig; [27]).

### Mineral analysis of skin discs

Mature ‘WA 38’ apples (146−147 DAFB) were selected for the different stages of GS development around lenticels, including asymptomatic lenticels from bagged apples (BL), intact lenticels (OKL), lenticels surrounded by a green halo (GHL), lenticels with initial green spot formation (GHGS) and green spots associated with necrosis as indicated by skin browning (BHL). Epidermal discs (ES) were punched and excised from the pedicel end of the fruit using a biopsy punch (10 mm diameter, Integra™ Miltex^®^, Avantor) and a razor blade. The thickness of the ES ranged from 1.0 to 1.5 mm. The ES were excised, immediately frozen in liquid nitrogen, and stored at −80°C until further processing. All samples (~2 g frozen material per treatment and rep and five rep per GS developmental stage) were freeze-dried until constant weight and then ground using an A11 basic analytical mill (IKA Works, Wilmington, NC, USA). The dried powder was sent to a commercial laboratory for mineral analysis (Best Test, Moses Lake, WA). The total N, P, K, Ca, Mg, and S were determined and expressed as ratios relative to dry mass (mg g^-1^ DM). The following mineral ratios were calculated: N:Ca, K/Ca, (K + Mg)/Ca and (K + Mg + N)/Ca.

### Data analysis and statistics

The data in the figure and tables are presented as means ± standard errors of means. Where not shown, error bars are smaller than data symbols. Data were subjected to correlation analysis (Proc Corr), linear (Proc Reg) and non-linear regression analysis (Proc Nlin) and analysis of variance (AOV; Proc GLM) or non-parametric tests (Proc Npar1way, Proc Multtest) using SAS (Version 9.1.4; SAS Institute, Cary, NC). Means were compared using Tukey’s studentized range test at P = 0.05, the Dunnett test at P = 0.05, or contrasts. Percentage data were arcsine transformed prior to AOV.

## Results

### Fruit growth and allometry

Fruit mass of ‘WA 38’ and its parent cultivars, ‘Enterprise’ and ‘Honeycrisp’, increased with time (Fig 1A). Up until 120 DAFB, the growth rate was highest for ‘WA 38’, followed by ‘Enterprise’ and ‘Honeycrisp’. ‘WA 38’ attained the maximum growth rate within about 80 DAFB, whereas the rate of growth of ‘Enterprise’ and ‘Honeycrisp’ kept increasing until about harvest (Fig 1A, inset). Fruit of ‘WA 38’ were longer than those of ‘Enterprise’ and ‘Honeycrisp’ (Fig 1B). The rate of increase of diameter between ‘WA 38’ and its parents was similar (Fig 1C). Fruit height/diameter ratio decreased in all cultivars up to 75 DAFB and then remained about constant (Fig 1D). Among the three cultivars, ‘WA 38’ had the highest height-to-diameter ratio from 100 DAFB to maturity, followed by ‘Enterprise’ and ‘Honeycrisp’.

Allometric analyses established that growth rates differed across regions in ‘WA 38’ (Fig 2; Table 1), ‘Enterprise’ (S3 Fig), and ‘Honeycrisp’ (S4 Fig). Generally, growth in length of the lobes in the calyx and the pedicel end region was hyperallometric relative to the growth in fruit diameter (slope of log plot > 1; Table 1), while that in core length was hypoallometric compared to growth in diameter (slope < 1; Table 1). In the remaining regions, growth was essentially isometric compared to the increase in fruit diameter (slope ~ 1; Table 1). The growth in length of the calyx lobes was more rapid in ‘WA 38’ compared to ‘Honeycrisp’ followed by ‘Enterprise’. In the pedicel end region, the length of the pedicel lobes of ‘Honeycrisp’ increased more rapidly than that of ‘WA 38’. In ‘Enterprise’, growth in length was more isometric than in the other two cultivars (Table 1).

### Position of vascular bundles

Transverse cross sections in the equatorial plane revealed that the position of petal and sepal bundles in asymptomatic ‘WA 38’, ‘Honeycrisp’ and ‘Enterprise’ were somewhat unique (Table 2). In all three cultivars, the petal bundles were more peripheral than the sepal bundles (S2 Fig). This was most evident for ‘WA 38’, followed by ‘Enterprise’ and ‘Honeycrisp’.

**Table 2:** Fruit radius and position of sepal and petal bundles in mature ‘WA 38’ and its parent cultivars ‘Enterprise’ and ‘Honeycrisp’ at 151 and 154 days after full bloom for ‘WA 38’ and ‘Honeycrisp’, and ‘Enterprise’, respectively. The position of the vascular bundles was expressed as a percentage of the equatorial fruit radius.

Cultivar	Fruit radius (mm)	Distance of bundles from center (% of radius)		
		Sepal (sb)	Petal (pb)	Sepal minus petal (sb-pb)
‘**WA 38**’	38.1 ± 0.6 a	43.2 ± 1.1 b	53.0 ± 0.9 b	−9.8 ± 0.7 c
‘**Honeycrisp**’	34.5 ± 0.9 b	51.9 ± 0.9 a	52.9 ± 1.0 b	−1.1 ± 0.7 a
‘**Enterprise**’	38.1 ± 0.8 a	51.4 ± 0.9 a	55.9 ± 0.6 a	−4.5 ± 0.8 b

Mean separation within columns by Tukey’s studentized range test, *P* = 0.05.

Within ‘WA 38’, petal bundles were slightly more peripheral in an equatorial cross-section of symptomatic fruit affected by green spots (BHL) than in bagged fruit (BL) or asymptomatic fruit (OKL), but this difference was not significant (Table 3). There was also no significant difference in the position of the sepal bundles. Accordingly, the difference in % radius between sepal and petal bundles was most negative for green spot-affected fruit (BHL in Table 3) as compared to that in bagged fruit (BL). In contrast, asymptomatic fruit (OKL) ranged between the other scenarios.

**Table 3:** Fruit radius and position of sepal and petal bundles in apples of ‘WA 38’ at 147 days after full bloom, which were not affected (OKL), and severely affected by green spots (BHL). Lenticels of bagged fruit (BL) were included as an additional control because early-bagged fruit did not develop green spot in past evaluations [11]. The position of the vascular bundles was expressed as a percentage of the equatorial fruit radius.

Stages	Fruit radius (mm)	Distance of bundles from center (% of radius)		
		Sepal (sb)	Petal (pb)	Sepal minus petal (sb-pb)
**BL**	39.2 ± 0.4 a	45.1 ± 0.7 a	50.8 ± 0.7 b	−5.7 ± 0.5 a
**OKL**	36.8 ± 0.6 b	46.9 ± 1.0 a	53.3 ± 1.0 ab	−6.4 ± 0.7 ab
**BHL**	37.7 ± 0.4 ab	47.1 ± 0.6 a	54.9 ± 0.5 a	−7.8 ± 0.4 b

Mean separation within columns by Tukey’s studentized range test, *p* = 0.05.

**Fig 3:**
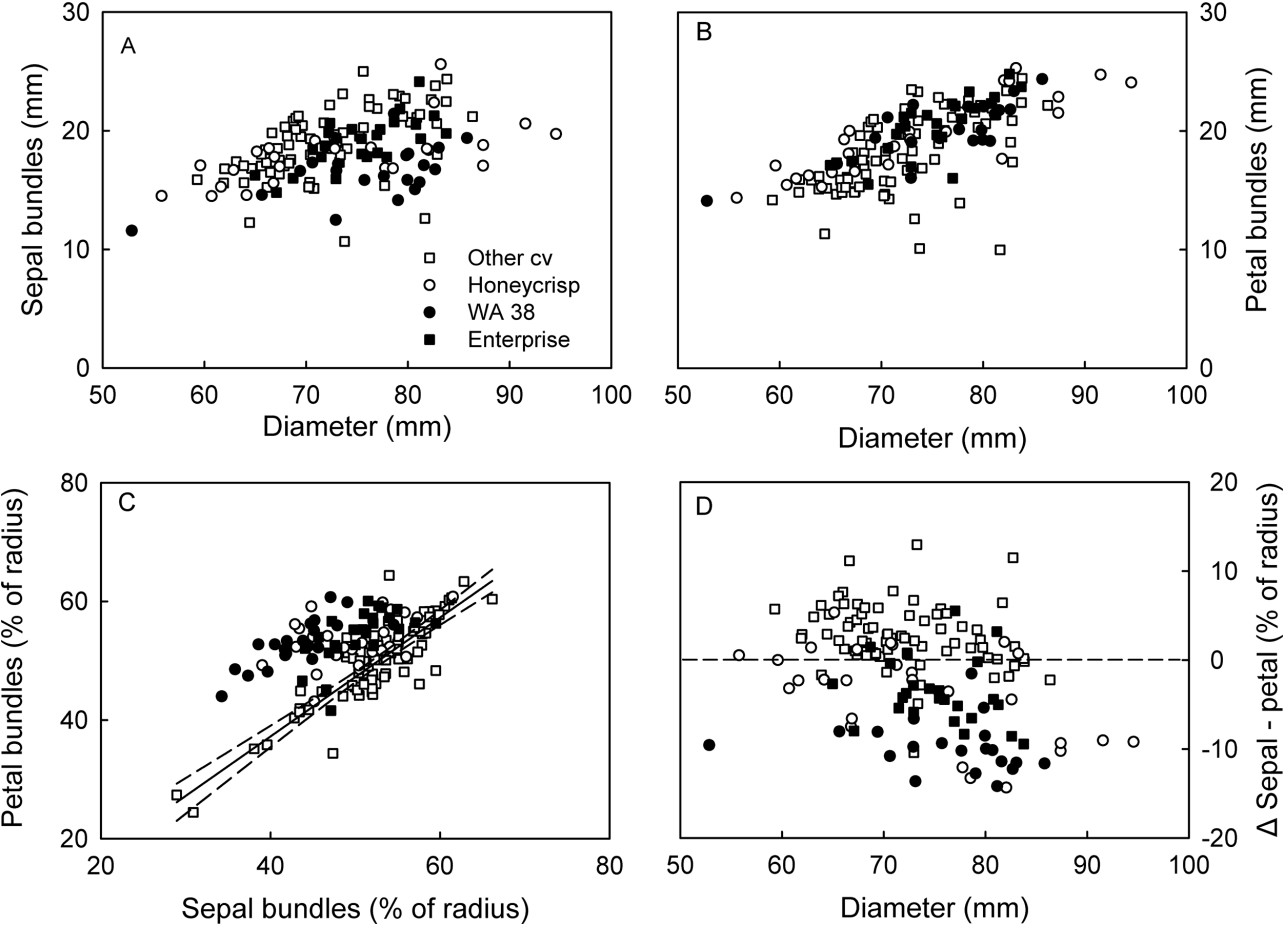
Relationship between the position of sepal bundles (A) and petal bundles (B) and fruit diameter in a range of apple cultivars (other cv), ‘WA 38’, ‘Enterprise’, and ‘Honeycrisp’. The cultivars used for comparison included ‘Ambrosia’, ‘Earlygold’, ‘Gala’, ‘Granny Smith’, ‘Idared’, ‘Jonastar’, ‘*Malus* 22’, ‘Newtown Pippin’, ‘Cripps Pink’, ‘Delicious’, ‘September Wonder’, and ‘WA 64’. **(C)** Relationship between the position of petal bundles and sepal bundles. The position of the vascular bundles was expressed as a percentage of the equatorial radius of the fruit. The regression line was fitted only through the data of the cultivar collection (r^2^ = 0.88***). **(D)** Difference between position of sepal and petal bundles plotted as a function of fruit diameter. For bundle position in individual cultivars, see Table 4. Data points represent individual fruit.

The distance of the sepal and the petal bundles from the center of the core increased for all cultivars as fruit diameter increased (Fig 3A and 3B). Expressing the distance of the bundles from the core as a percentage of the fruit radius also revealed a positive relationship between the distances of the petal and the sepal bundles (Fig 3C). If a cultivar had central or peripheral arrangements of sepal bundles, then it has the same arrangement of petal bundles. This relationship was highly significant for the selection of cultivars (Fig 3C, r^2^ = 0.88***). In 10 out of 12 cultivars from the cultivar collection, the sepal bundles were more peripheral than the petal bundles (Table 4). The two exceptions, ‘Idared’ and ‘*Malus* 22’, had about equal radii of sepal and petal bundles from the core. However, ‘WA 38’, ‘Honeycrisp’ and ‘Enterprise’ clearly deviated from this relationship (Fig 3C). In the latter, the petal bundles were markedly more peripheral than the sepal bundles. This was most evident in ‘WA 38’ (mean radii difference sepal minus petal −9.8 ± 0.7%, Table 2), followed by ‘Enterprise’ (mean difference sepal minus petal −4.5 ± 0.8%) and ‘Honeycrisp’ (mean difference sepal minus petal −1.1 ± 0.7%) (Fig 3D, Table 2).

**Table 4:** Fruit radius and position of sepal and petal bundles in apples of selected cultivars. The position of the vascular bundles was expressed as a percentage of the equatorial fruit radius.

Cultivar	Fruit radius	Distance of bundles from center (% of radius)		
	(mm)	Sepal (sb)	Petal (pb)	Sepal minus petal (sb-pb)
**‘Ambrosia’**	33.5 ± 0.5 cb	51.4 ± 0.8 bdc	45.9 ± 0.6 b	5.5 ± 0.6 ab
**‘Earlygold’**	32.6 ± 0.7 d	54.8 ± 1.2 ab	47.7 ± 0.7 b	7.1 ± 0.9 a
**‘Gala’**	34.8 ± 0.5 cd	59.6 ± 1.5 a	57.1 ± 1.3 a	2.4 ± 0.7 abc
**‘Granny Smith’**	37.6 ± 1.2 abc	59.6 ± 0.8 a	58.7 ± 1.2 a	0.9 ± 0.6 bc
**‘Idared’**	40.6 ± 0.9 a	50.9 ± 0.8 cb	52.5 ± 0.7 ab	−1.6 ± 0.8 c
**‘Jonastar’**	35.9 ± 0.5 cb	51.3 ± 0.3 bc	47.0 ± 0.8 b	4.3 ± 0.8 ab
**‘*Malus* 22’**	38.2 ± 0.8 abc	56.3 ± 1.4 cb	57.9 ± 1.7 a	−1.6 ± 1.9 c
**‘Newton Pippin’**	33.7 ± 0.9 d	50.5 ± 1.6 cb	44.9 ± 2.6 b	5.6 ± 0.7 ab
**‘Cripps Pink’**	33.2 ± 0.6 d	46.1 ± 1.8 cd	45.2 ± 2.1 b	0.9 ± 0.7 bc
**‘Delicious’**	34.9 ± 0.7 cd	54.1 ± 0.8 ab	51.5 ± 0.6 ab	2.6 ± 0.7 abc
**‘September Wonder’**	39.1 ± 0.8 ab	56.4 ± 0.7 ab	52.7 ± 1.6 ab	3.6 ± 1.8 ab
**‘WA 64’**	38.4 ± 1.0 abc	39.3 ± 3.3 d	35.9 ± 3.4 c	3.4 ± 0.8 abc

Mean separation within columns by Tukey’s studentized range test, P = 0.05.

### Functionality of petal and sepal bundles

**Fig 4:**
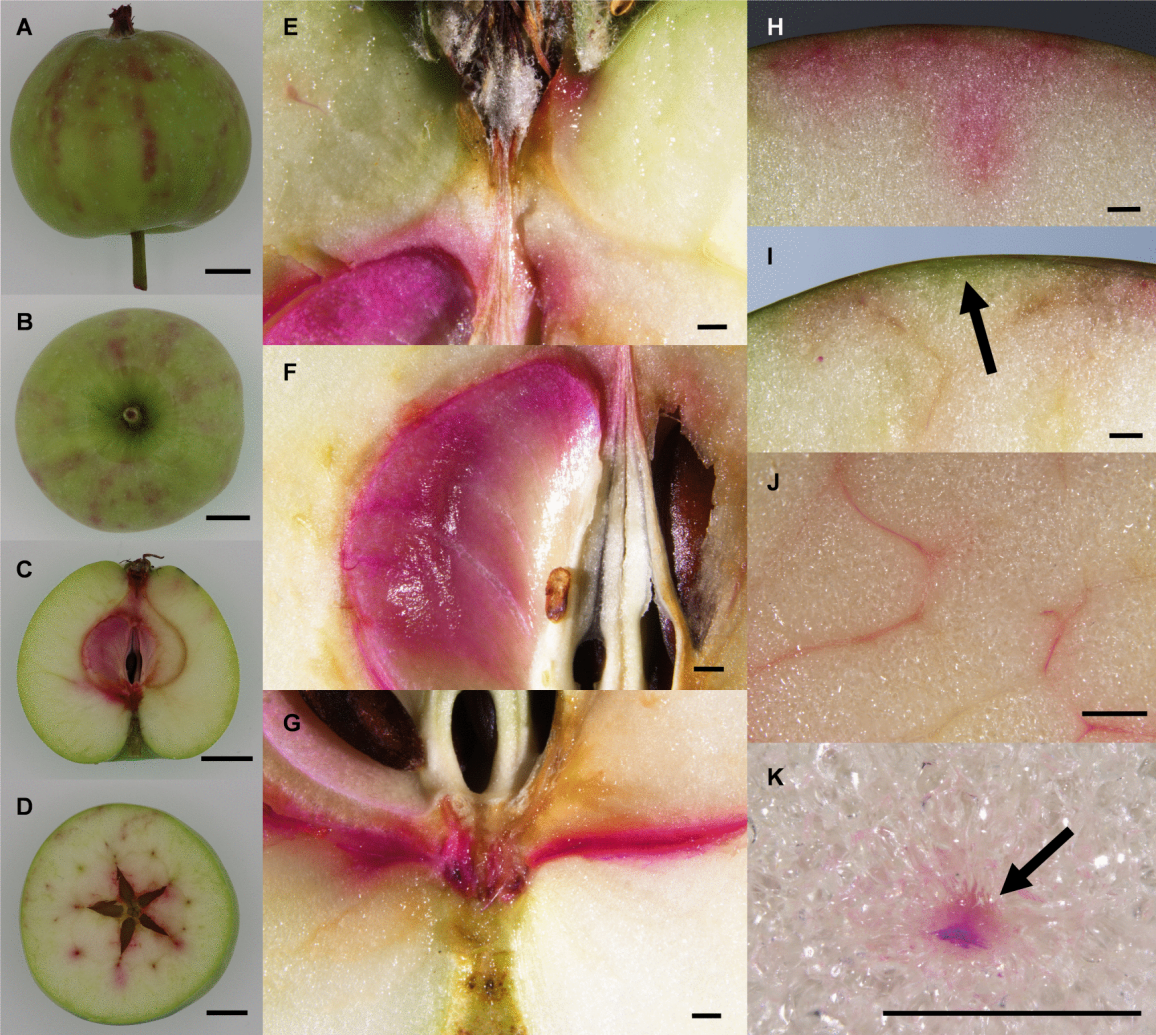
Representative images of immature ‘WA 38’ apple after 22 h of feeding acid fuchsin to the pedicel. **(A)** Side view. **(B)** Bottom view (pedicel facing the camera). **(C)** Longitudinal cross-section. **(D)** Latitudinal cross-section. **(E to K)** Detail of longitudinal cross section showing calyx cavity **(E)**, seed chambers **(F)** and pedicel cavity **(G)**. **(H,** **I)** Cross sections through skin. Arrow in I indicates position of lenticel. There is no vascular connection to the lenticel. **(J)** Tangential section underneath skin showing stained network of minor veins. **(K)** Cross-section perpendicular to fruit surface showing a stained minor vein. Images were taken from secondary bloom fruit that had a fresh mass similar to fruit of primary bloom at 63 DAFB (45.3 g mass). Scale bar in A to D is 10 mm, and E to K is 1 mm.

Feeding acid fuchsin to the pedicels of immature ‘WA 38’ resulted in stained longitudinal traces from the pedicel end to the calyx that were visible on the fruit surface (Fig 4A and 4B). The traces corresponded to the position of the sepal and petal bundles of ‘WA 38’. Cross sections revealed stained seed cavities and a stained vasculature within the core (Fig 4C, 4D, 4F and 4G), while most of the bundles at the distal calyx end were not stained (Fig 4E). The skin had a dense network of minor veins with numerous anastomoses immediately below the fruit surface (Fig 4H–4K). These minor veins were stained with acid fuchsin. The stain occasionally leaked into the surrounding tissue as indexed by a diffuse pattern of fuchsin in the vicinity of the minor veins (Fig 4H). No direct vascular connection was detected between the lenticels and the minor veins in the dermal skin beneath the lenticels of ‘WA 38’ (see arrow in Fig 4I).

**Fig 5:**
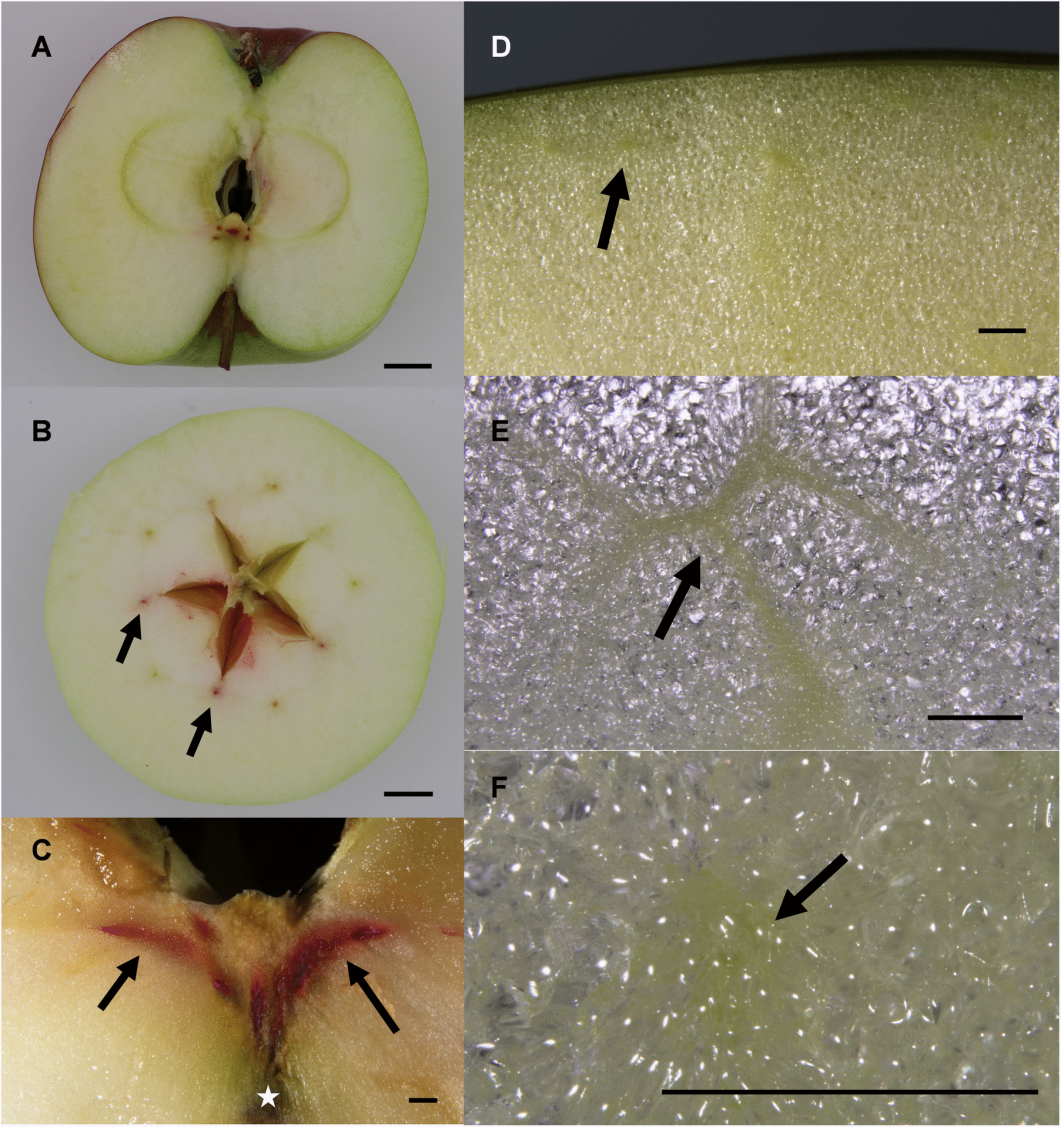
Mature ‘WA 38’ apple (144 DAFB) after 22 h of feeding acid fuchsin to the pedicel. **(A)** Longitudinal cross-section. **(B)** Latitudinal cross-section with two sepal bundles stained (see arrows). **(C)** Detail of pedicel end with pedicel cavity showing two vascular bundles that have their proximal end stained (see arrows). **(D)** Cross-section through skin showing a network of minor veins without staining. **(E)** Tangential section underneath skin showing a network of minor veins without staining. **(F)** Cross-section perpendicular to fruit surface showing a minor vein without staining. Scale bar in A and B 10 mm, C to F 1 mm.

In contrast, in mature ‘WA 38’, there was very little staining of vascular bundles and seed cavities (Fig 5A–5C). The staining was limited to the major vascular bundles below, i.e., proximal to the seed cavity (closer to the pedicel) (Fig 5C). Accordingly, the minor vein network close to the fruit surface remained entirely unstained (Fig 5D–5F).

There was no significant difference in the number of stained sepal bundles between immature and mature ‘WA 38’, but immature ‘WA 38’ had a higher number of stained and, hence, functional petal bundles as compared to mature fruit (Table 5). Immature and mature ‘WA 38’ had the same inflow rates of fuchsin solution despite a sixfold higher mass of the mature fruit (Table 5). Interestingly, bagged ‘WA 38’ had more functional petal bundles than non-bagged fruit. The rates of inflow of fuchsin did not differ. There was no difference in vascular functionality between symptomatic and asymptomatic ‘WA 38’ (data not shown).

**Table 5:** Effect of fruit development and effect of bagging on functionality of sepal and petal bundles in ‘WA 38’. The experiment on the effect of fruit development was studied at 139 and 144 days after full bloom (DAFB) and on the impact of bagging at 153 and 154 DAFB. For the effect of fruit development, fruit from secondary bloom was used. This fruit had a mass equivalent to the mass of primary bloom fruit at 65 DAFB. The functionality of bundles was assessed by feeding pedicels with acid fuchsin and quantifying the number of stained vascular bundles and total vascular bundles in the equatorial plane of the fruit at 22 h after feeding.

Treatment		Mass (g)		Flow	Sepal bundles (No per)				Petal bundles (No per fruit)			
				(µl in 22 h)	Stained		Total		Stained		Total	
**Effect of fruit development**												
**Young**		45.3 ± 4.7 b		263.0 ± 37.0 a	2.6 ± 0.4 a		5.0 ± 0.0 a		3.0 ± 0.6 a		5.9 ± 0.3 a	
**Mature**		224.5 ± 13.2 a		180.4 ± 27.1 a	2.9 ± 0.6 a		5.0 ± 0.0 a		0.5 ± 0.2 b		6.0 ± 0.3 a	
**Effect of bagging**												
**Bagged fruit**	212.3 ± 9.7 b		135.3 ± 14.6 a			3.0 ± 0.3 a		5.3 ± 0.1 a		1.3 ± 0.3 a		6.1 ± 0.2 a
**Non-bagged fruit**	258.9 ± 8.5 a		144.1 ± 14.8 a			2.5 ± 0.3 a		5.1 ± 0.1 a		0.4 ± 0.1 b		6.0 ± 0.2 a

N=12 to 14 for fruit development, n=24 for bagged fruit.

Data represent means ± SE.

Mean comparisons within experiments and within columns by Tukey’s studentized range test, P = 0.05.

**Fig 6:**
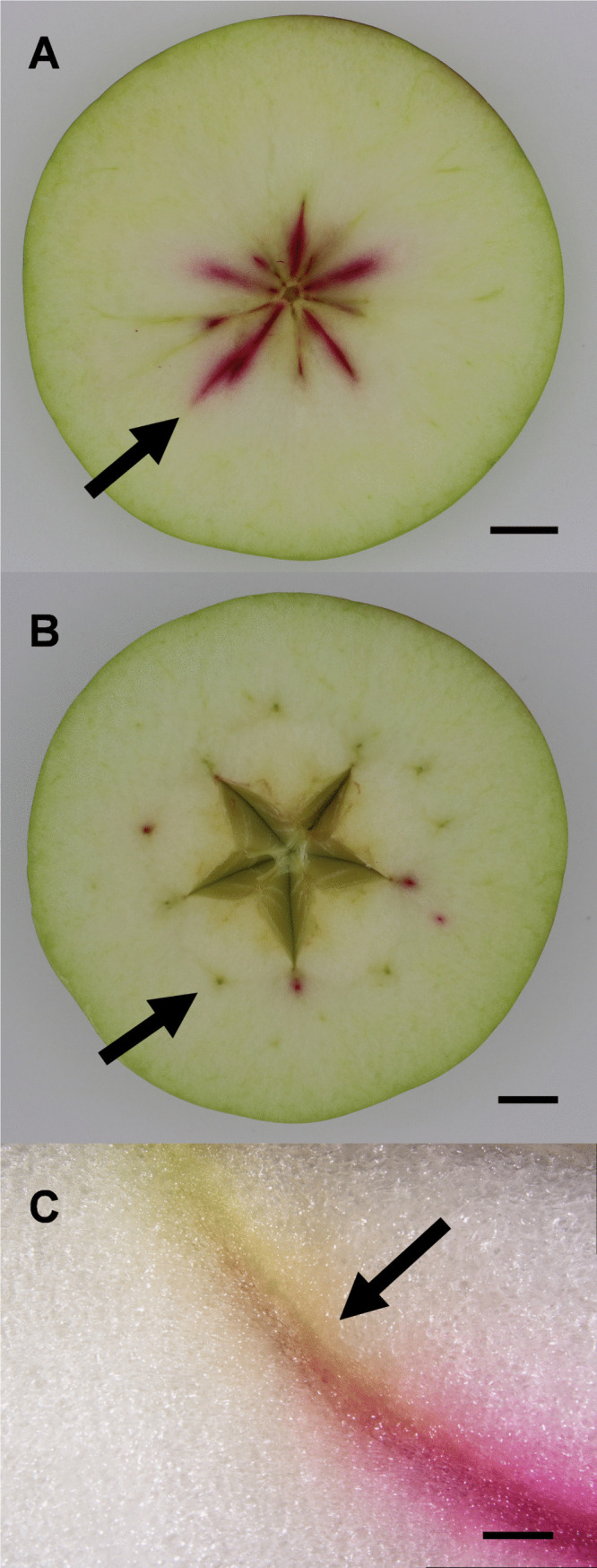
Distal (A) and proximal surfaces (B, mirror image) of a slice taken as a transversal cross-section through a mature ‘WA 38’ apple from the base of the seed chamber after 22 h of feeding acid fuchsin. The slice was 5 mm thick. Note that all petal bundles are stained on the proximal surface (facing the pedicel end, A) but not on the distal surface (facing the calyx, **B**). Petal bundles are located between the seed chambers, and sepal bundles are in front of the seed chambers (See S2 Fig). Arrow indicates the same petal bundle on proximal and distal surface. **(C)** Detail view of the rupture zone of the very same petal bundle, revealing fuchsin staining at the proximal and lack of stain at the distal surface (see arrow) of the same slice.

Further cross sections were prepared to localize the point of vascular discontinuity at the proximal end of the seed cavity of mature ‘WA 38’ fruit. Microscopy revealed that the loss of continuity of the xylem must have occurred at the base of the seed cavity (Fig 6A–6C). The thickness of the representative fruit slice depicted in Fig 6A and 6B was 5 mm. On the proximal surface of the slice, sepal and petal bundles were stained, whereas on its distal surface, only two out of five sepal bundles and only one out of six petal bundles were stained. Thus, the vascular continuity of the petal bundles was disrupted within the slice. Further sectioning localized the loss of functionality to the proximal end of the seed cavity, where the petal bundles bent towards the distal end, in the direction of the calyx (Fig 6C).

### Mineral analysis of skin discs affected by GS

Skin discs of bagged fruit had the lowest N and Ca, but the highest K dry mass ratio (Table 6). For the unbagged fruit, the dry mass ratios of N and Mg increased as GS development progressed from the OKL to the BHL stage, whereas the dry mass ratio of K decreased (Table 6). There was no consistent effect of GS development on the dry mass ratio of Ca. The N/Ca ratio increased as GS development progressed. However, the ratios of K/Ca and (K + Mg)/Ca tended to decrease in green-spot-affected skin, whereas (K + Mg + N)/Ca remained unaffected. Interestingly, bagged fruit in the BL stage had the highest ratios of K/Ca, (K + Mg)/Ca and (K + Mg + N)/Ca (Table 6).

**Table 6:** Effect of different stages of green spot development on the mineral content of fruit skins surrounding lenticels on mature ‘WA 38’ apples at 146-147 DAFB. The mineral composition was expressed as mineral/dry mass ratios (DM, top) and as ratios (bottom). The stages investigated were unbagged normal lenticel (OKL), unbagged lenticel surrounded by a green halo (GHL), unbagged lenticel with a green halo and a developing green spot (GHGS), and unbagged lenticel with a fully developed green spot and associated cell death (BHL). For comparison, the characteristics of lenticels of bagged fruit are included (BL).

Stages	Mineral dry mass ratio (mg g^-1^ DM)				Ratios			
	N^1^	K^1^	Ca^1^	Mg^2^	N/Ca^1^	K/Ca^2^	(K + Mg)/Ca^2^	(K + Mg + N)/Ca^2^
**BL**	4.02 ± 0.15 c	10.49 ± 0.33 a	0.53 ± 0.03 b	0.98 ± 0.02 b	7.7 ± 0.4 ab	20.0 ± 1.6 a	21.9 ± 1.7 a	29.5 ± 2.1 a
**OKL**	4.52 ± 0.24 bc	9.36 ± 0.69 a	0.81 ± 0.07 a	0.99 ± 0.05 b	5.7 ± 0.3 c	11.6 ± 0.4 bc	12.9 ± 0.4 bc	18.5 ± 0.7 b
**GHL**	4.82 ± 0.27 bc	9.60 ± 0.50 a	0.75 ± 0.08 ab	0.99 ± 0.06 b	6.6 ± 0.4 bc	13.2 ± 1.0 b	14.5 ± 1.1 b	21.1 ± 1.5 b
**GHGS**	5.19 ± 0.22 b	6.80 ± 0.21 b	0.81 ± 0.02 a	1.07 ± 0.05 b	6.5 ± 0.4 bc	8.4 ± 0.2 c	9.8 ± 0.2 c	16.2 ± 0.4 b
**BHL**	6.70 ± 0.33 a	7.06 ± 0.22 b	0.75 ± 0.06 ab	1.64 ± 0.05 a	9.0 ± 0.3 a	9.5 ± 0.5 bc	11.8 ± 0.7 bc	20.8 ± 0.9 b

1 Analysis of variance and mean separation within columns by Tukey’s Studentized range test, P = 0.05, n = 5.

2 Non-parametric test using Proc Multtest and mean separation by contrasts, P = 0.05, n = 5.

## Discussion

### Rapid growth in the pedicel end region and the arrangement and functionality of cortical vascular bundles are related

**‘**WA 38’, **‘**Honeycrisp**’** and **‘**Enterprise**’** are large-fruited apple cultivars that exhibit high growth rates throughout their development. This is particularly the case under the growing conditions of Washington State, where light intensities and daytime temperatures are high [28], often with nighttime cooling [11]. These result in water vapor pressure deficits (WVPD) that markedly oscillate between day and night. The WVPD is high during daytime, but decreases at night [19,29]. The WVPD is the driving force for transpiration; fluctuations in WVPD translate into oscillations of fruit diameter [19]. During daytime, apple fruit diameter typically decreases due to transpiration and, possibly, backflow of water via the xylem from the fruit to the tree [19]. In contrast, during nighttime, fruit diameter increases, reaching its maximum around dawn [19]. Thus, growth rates on an hourly basis oscillate markedly throughout the day, leading to peak growth rates much larger than those calculated as the first derivative of the cumulative increase in mass during fruit development over the whole season. Diurnal growth rates, measured as changes in fruit diameter, have been reported for ‘Cox’s Orange’ and ‘Gala’ apples [19]; however, comparable data are unavailable for ‘WA 38’, ‘Honeycrisp’, or ‘Enterprise’. Given their larger fruit size and the environmental conditions in Washington State, we expect these cultivars to exhibit substantially greater diurnal variation in fruit diameter and surface area than the smaller-fruited ‘Cox’s Orange’ and ‘Gala’.

Growth rates vary not only on a temporal scale but also on a spatial scale within an apple. Allometry revealed that growth in length is particularly rapid in the proximal (pedicel end) and the distal (calyx lobe) region of ‘WA 38’ and ‘Honeycrisp’. In contrast, longitudinal growth is slow in the equatorial core area. High rates of growth may result in xylem rupture, whereas the phloem typically remains functional [20]. Xylem vessels are sclerenchymatic and dead when functional. Their secondary cell walls are rigid and not extensible. Phloem cells are mostly alive with primary extensible cell walls. Fibers that occur in xylem and phloem are dead at maturity and have rigid secondary cell walls. Growth subjects xylem and phloem to an *in vivo* tensile test. At high growth rates, one would expect failure of rigid, less extensible structures like xylem vessels, and fibers of xylem and phloem, whereas the more extensible sieve tubes of the phloem remain intact [30]. Growth-induced rupture of the cortical xylem was demonstrated for a number of fruit crop species, including the apple cultivars ‘Braeburn’ and ‘Granny Smith’ [20] and ‘Honeycrisp’ [21]. Interestingly, the petal bundles in ‘WA 38’ ruptured more frequently than the sepal bundles. This was unexpected but likely related to the unusual arrangement of bundles. In ‘WA 38’, petal bundles were more peripheral than sepal bundles. This was also observed in ‘Honeycrisp’ and ‘Enterprise’, although to a lesser extent. In most other apple cultivars, however, sepal bundles are more peripheral than petal bundles; while adnate with each other and the gynoecium of this epigynous fruit, the corolla is peripheral to the corona (see Fig 13 in MacDaniels, 1940 [27]). Also, the petal bundles in fruit affected by GS (stage BHL) were more peripheral and the difference in radii between sepal and petal bundles was larger as compared to asymptomatic fruit (stage OKL) or to bagged fruit (BL). The unusual arrangement of bundles in ‘WA 38’ subjects the more peripheral petal bundles to larger stress than the sepal bundles. The unusual arrangement of the bundles may be caused by excessive growth of the core between two neighboring carpels. This argument explains the unusual bundle arrangements in ‘WA 38’, ‘Honeycrisp’ and ‘Enterprise’. This also explains the higher frequency of functional loss in petal bundles compared to sepal bundles in ‘WA 38’.

Interestingly, bagged fruit had more functional bundles than non-bagged fruit. Furthermore, bagged fruit had the smallest difference in radii between sepal and petal bundles. Also, bagged ‘WA 38’ showed consistently smaller gaping than non-bagged ‘WA 38’ [31]. In addition, bagged ‘WA 38’ had the lowest frequency of cracked lenticels compared to non-bagged fruit, although this effect was not significant [31]. These observations suggest lower tissue stress and strain in the bagged fruit. The following argument accounts for lower stress and strain. Bagging altered the microclimate around the fruit, reducing temperature and increasing relative humidity, thereby lowering WVPD [11]. The lower WVPD and restricted air movement inside the bag reduce transpiration and dampen diurnal fluctuations in fruit diameter, thereby decreasing growth-related stress. The lower WVPD of bagged ‘WA 38’ fruit could also explain the higher infiltration of lenticels with acridine orange observed by [31]. Bagging decreased cuticle deposition in ‘WA 38’ [31] and also in mango [32]. However, the higher number of functional petal bundles in bagged ‘WA 38’ fruit was insufficient to prevent the Ca/dry mass ratio of bagged fruit from decreasing compared to non-bagged fruit. Despite a lower Ca inflow, bagged fruit rarely developed GS or BP. These data confirm a report that a low Ca/dry mass ratio did not necessarily lead to higher GS or BP incidences [33]. The physiological mechanism that decreased the incidence of GS in this and our earlier study is unknown [11,12].

### Relationship between fruit growth, functionality of vascular bundles and mineral content

Little change in mineral content was observed as green spot (GS) progressed from OKL to GHGS stages. The absence of a consistent effect of xylem dysfunction on mineral composition is somewhat surprising, particularly for calcium and potassium. A decline in the Ca/dry mass ratio and an increase in the K/dry mass ratio were expected as GS development advanced. Such effects are typically exacerbated by high light intensity and elevated temperatures during growth [34], conditions to which ‘WA 38’ was exposed in Washington State. However, this was not observed in the present case. In fact, compared to asymptomatic bagged fruit, the various stages of GS development had even more Ca than the bagged fruit. The K/dry mass ratio was higher in bagged fruit and the OKL and GHL stages of GS development of non-bagged fruit compared to the GHGS and BHL stages. Further, the K/dry mass ratio decreased as GS development progressed to the GHGS and BHL stages. However, the following arguments must be considered: First, among the lenticels investigated, only those at the OKL stage lacked visible symptoms, and it is not known whether lenticels at the OKL stage will eventually develop into GHL, GHGS, and BHL. Second, there was no difference in functionality of vascular bundles between mature symptomatic fruit (stage BHL) and fruit without any GS symptoms (stage OKL). Third, bagging clearly reduced/eliminated green spots, but bagging altered the microclimate around the fruit. As a consequence, the Ca/dry mass ratio was lowered compared with non-bagged fruit despite having more vascular bundles compared to asymptomatic fruit (OKL stage). Thus, bagging was not a suitable control for the skin mineral analysis because of these confounding factors. These arguments demonstrate that the stages of GS development may not necessarily reflect differences in mineral content.

### A hypothetical mechanism of green spot formation in ‘WA 38’

In many respects, green spot (GS) in ‘WA 38’ closely resembles bitter pit (BP) in ‘Honeycrisp’. This similarity is not unexpected, given that ‘WA 38’ is a direct offspring of ‘Honeycrisp’, and other ‘Honeycrisp’ progenies have also been observed to develop similar skin disorders [22]. Other than some etiological differences—such as the developmental stage at which symptoms appear and the typical location of symptoms on the fruit—these observations suggest a genetic component contributing to the susceptibility of ‘Honeycrisp’ progenies to BP-like disorders such as GS. That BP and GS may share a common mechanism is based on the following arguments:

#### Similarity of visual symptoms.

In GS, symptoms typically occur on the fruit shoulder, whereas in BP, they are more commonly located near the calyx end. The visual symptoms of GS at their final stage in ‘WA 38’ are similar to those of BP in ‘Honeycrisp’, with the primary differences being the spatial distribution along the pedicel/calyx axis [11] and the frequent association of GS with lenticels [31]. These distinctions may be explained by variation in the spatial pattern of xylem rupture or by differential calcium (Ca) binding, potentially caused by local depletion of apoplastic free Ca needed to stabilize membranes [15,35–37].

#### Similarity of growth pattern and rupture of bundles.

A number of the conditions recently identified that cause BP in ‘Honeycrisp’ are identical to those causing GS in ‘WA 38’. Specifically, both cultivars bear large fruit and consequently have high growth rates (Fig 1). The incidences of BP in ‘Honeycrisp’ and of GS in ‘WA 38’ are positively related to fruit size in this and earlier studies [31,37,38].

Factors that increase vigor and rate of fruit growth, such as low crop load, also increased GS frequency in ‘WA 38’ [18,31] and BP frequency in ‘Honeycrisp’ [39]. Similarly, decreased vigor, for example, due to deficit irrigation, decreased the incidence of BP in ‘Honeycrisp’ [38].

Also, GS in ‘WA 38’ develops under high temperatures and light conditions that favor high growth rates, such as those prevailing in Washington State, USA and South Tyrol, Italy. There are no reports on GS from cooler environments with lower light intensities, like northern Germany, where we expect growth rates to be much lower.

As argued above, high growth rates, particularly at the pedicel end of both ‘WA 38’ and ‘Honeycrisp’ and, to a lesser extent, ‘Enterprise,’ caused marked longitudinal and tangential growth stress and strain. The unusual arrangement of petal bundles aggravates stress and strain. These, in turn, result in rupture of the xylem in developing ‘WA 38’ in our study and in ‘Honeycrisp’ in studies by Griffith and Einhorn (2022) [21]. Rupture of the xylem impairs Ca translocation and results in nutrient and hormonal imbalances that are suspected to be causal in BP [21] and, possibly, GS.

#### Mineral content.

Given the visual symptom overlap between GS and BP, both disorders are likely to share a similar physiological basis. Bitter pit is generally considered to result from local Ca deficiency.

However, there was no consistent difference in Ca/dry mass ratio between different stages of GS development in our study or between fruit with or without GS in the study by Sallato et al. (2021) [18]. Also, there was no difference in functionality of vascular bundles between symptomatic fruit (stage BHL) and fruit without any GS symptoms (stage OKL). Comparing the Ca/dry mass ratio of ‘WA 38’ with those reported for other apple cultivars revealed that the Ca/dry mass ratio of ‘WA 38’ fruit was well within the range reported as sufficient in other apple cultivars [40–42]. Further, the Ca/dry mass ratio was above that reported for BP-affected ‘Honeycrisp’ [43]. Interestingly, BP incidence is only weakly related to Ca level in ‘Honeycrisp’ apples and in those of other cultivars, and only in some cases [14,17]. Fruit having Ca deficiency often has the same or even higher total Ca content than healthy fruit grown under the same growing conditions [14,21,41,44,45].

The lack of clear relationships may be due to the greater complexity of Ca physiology in apples. Ca accumulates in various inter- and intracellular pools, where it performs diverse functions [15,21,36]. Cell wall-bound Ca makes up the largest Ca pool, followed by vacuolar Ca. Only minor amounts of Ca are free in the apoplast. Calcium from the free apoplastic pool plays a role in stabilizing cellular membranes and is a key factor in the development of bitter pit (BP). However, this free apoplastic calcium competes with calcium bound to the cell wall and stored in the vacuole, potentially limiting its availability for membrane stabilization. Changes in cell wall-bound Ca levels may result from increased expression or activity of cell wall-degrading enzymes, such as pectin methylesterases (PMEs). As a consequence, the binding site for Ca increases and increased binding depletes free apoplastic Ca available for stabilizing membranes [15,46]. Similarly, increased expression and/or activity of cation/H^+^ exchangers, so-called CAXs, responsible for Ca^2+^/H^+^ exchange at the vacuole tonoplast, and Ca-ATPases may increase Ca accumulation in storage organelles, resulting in further depletion of the apoplastic Ca pool, localized membrane breakdown, and BP development [15,36]. Further, malic acid stored in the vacuole of apple fruit may form precipitates of Ca-malate. This complex formation also decreases the pool of free Ca available to stabilize membranes [46]. Support for the above explanations comes from a recent study by Islam et al. (2022) [47]. The study provides evidence for relationships among PME activity, pectin de-esterification level, cell wall-bound Ca, and the apoplastic free Ca concentration required to maintain cell membrane integrity and reduce BP development [47]. Decreased PME activity increased free apoplastic Ca, accounting for the effect of rootstocks on BP incidence in ‘Honeycrisp.’ Also, the occurrence of the green halo (GH) around lenticels during early GS formation is consistent with a lack of Ca associated with membrane. The increase in membrane permeability then causes water to leak into the formerly gas-filled-cell wall space, resulting in the water soaked appearance of the GH around lenticels. Interestingly, similar observations are reported for early stage of BP development [15].

Currently, there is no available information for ‘WA 38’ regarding the distribution of calcium across different Ca pools, nor on the activity of cell wall–degrading enzymes such as pectin methylesterase (PME), or the activity and expression of calcium transporters—all of which would compete with cellular membranes for available calcium [46].

Further complications arise from interactions among the individual Ca pools and other minerals. In our experiment, the highest N levels were recorded in the skins of ‘WA 38’ with severe BHL symptoms of GS. Coincidentally, high N levels were also reported in BP [46,48]. Also, the highest levels of Mg in ‘WA 38’ were measured in skins having most severe GS. High levels of Mg and K were also reported for BP [46,48]. These elements compete with Ca for membrane uptake and binding sites, potentially leading to localized Ca deficiency [15]. Based on the above arguments, a simple relationship between total Ca and the incidence of GS in ‘WA 38’ or of BP in ‘Honeycrisp’ may not be expected.

## Conclusions

Our data indicate that green spot (GS) in ‘WA 38’ shares similar symptoms and certain etiological features with bitter pit (BP) in ‘Honeycrisp.’ In both cultivars, the high rate of fruit growth, particularly in the proximal pedicel region of ‘WA 38’ and the arrangement of their vascular bundles could result in dysfunctional xylem. This may cause imbalances in Ca delivery to the fruit. Although the total Ca/dry mass ratio of ‘WA 38’ appears sufficiently high, these imbalances may arise between competing Ca pools within the fruit. Specifically, the pool of free apoplastic Ca, which is responsible for stabilizing membranes, is causal in BP of ‘Honeycrisp’ and, possibly, in GS of ‘WA 38’ [15,49]. This pool competes with vacuolar Ca and cell wall-bound Ca, which are several-fold larger than that of apoplastic free Ca [36,46]. At present, the mechanism suggested herein is hypothetical. To confirm the role of Ca in GS in ’WA38’, further studies are needed. These should focus on (1) the developmental time course of change in Ca transport capacity of the xylem and the effect of growth rate thereon, (2) a quantification of the pool of apoplastic free Ca to critically test the hypothetical role of Ca in GS’WA 38’ fruit that would provide evidence for a close relationship between the rates of xylem rupture and fruit growth rate. Until the hypothesis of a Ca-related mechanism of GS formation is confirmed, alternative explanations should also be considered. For example, leaves infected with fungi develop "green islands" where senescence is retarded in the vicinity of the site of infection [50]. These "green islands" are due to the production of cytokinins that locally retard chlorophyll breakdown. The resulting "green islands" bear some similarity to GS of ‘WA 38.’ An involvement of microorganisms would also explain the association of GS and lenticels [11]. Lenticels represent openings in the fruit surface that bypass the cuticle as a penetration barrier for pathogen infection. To our knowledge, there is no indication of an association between GS and microorganisms, viruses, or insects, and the role of cytokinins has not been investigated to date. We expect that, if involved, this should have been detected in our earlier metabolomics study of GS-affected ‘WA 38’ [12]. Also, skin properties of ‘WA 38’ appear not to be causal in the formation of GS [31].

Based on the hypothetical mechanism of GS formation outlined above, field studies to develop countermeasures should focus on reducing the rate of fruit growth in ‘WA 38’ under high-light and high-temperature conditions. A lower rate of fruit growth is likely to decrease, or possibly delay, the rate at which the xylem becomes dysfunctional. In addition, measures that (1) stimulate xylogenesis and decrease the stiffness of the xylem such as the application of IAA or NAA [10], (2) reduce vigor [49] and (3) alter Ca partitioning (application of prohexadione-Ca, ABA; [51–53]) may also be beneficial in reducing the incidence of GS [10,54,55]. According to Griffith et al. (2021) [55], preventing membrane breakdown by the enzyme phospholipase could be an additional target for controlling BP and, hence, potentially GS. There is evidence that this may be achieved by spray application of hexanal [56], deficit irrigation [38,41], root pruning, defoliation [14,48], and the support of high crop loads will decrease the incidences of BP and, most likely, GS by increasing Ca translocation to the fruit [37]. From the above, it is also clear that a low fruit set [14,37,57], fertilization with N and/or K [10,14,37,41], or the application of GA [40] are likely to increase the incidence of GS and hence, are counterproductive.

## Supporting information

S1 Fig Relationship between fruit volume calculated from fruit diameter and fruit fresh mass of developing ‘WA 38’ (A), ‘Enterprise’ (B), and ‘Honeycrisp’ apple (C). (TIF)

S2 Fig (A) Schematic of the cross-section of ‘WA 38’ indicating the position of petal bundles (pb), sepal bundles (sp), relative to the seed chamber (sc). (B) Cross-section of ‘WA 38’ in the equatorial plane showing petal and sepal bundles and the seed chamber. Sepal bundles are located at the periphery of the seed chambers, while petal bundles are located between the seed chambers. Note the irregularity in the bundle number for ‘WA 38’. ‘WA 38’ fruit, however, often has excess bundles (for example, see Table 5 where the mean number of petal bundles was 6). In addition, in ‘WA 38’, the petal bundles are more peripheral than the sepal bundles. The ‘WA 38’ example shown in frame B has six sepal bundles and six petal bundles. The red color is due to acid fuchsin fed to the pedicel. For details, see materials and methods.
(TIF)

S3 Fig Allometric relationships between different longitudinal measures of developing ‘Enterprise’ apples. (A) Sketch of longitudinal section and dimensions identified. (B-H) Allometric relationships between the fruit center diameter and the maximum core diameter (B), the thickness of the flesh (C), the fruit diameter at the core base (D), total fruit length (E), the length of the pedicel lobes (F), the core length (G) and the length of the calyx lobs (H). All dimensions are log-transformed. For regression equations, see Table 1.
(TIF)

S4 Fig Allometric relationships between different longitudinal measures of developing ‘Honeycrisp’ apples. (A) Sketch of longitudinal section and dimensions identified. (B-H) Allometric relationships between the fruit center diameter and the maximum core diameter (B), the thickness of the flesh (C), the fruit diameter at the core base (D), total fruit length (E), the length of the pedicel lobes (F), the core length (G) and the length of the calyx lobes (H). All dimensions are log-transformed. For regression equations, see Table 1.
(TIF)

S1 File This is the Excel File containing the raw data used to calculate means and standard errors for Tables and Figures, including the figures of the supplemental information. (XLSX)
